# Sol–Gel Engineered MXene/Fe_3_O_4_ as an Efficient Mediator to Suppress Polysulfide Shuttling and Accelerate Redox Kinetics

**DOI:** 10.3390/gels11120959

**Published:** 2025-11-28

**Authors:** Zhenzhen Shan, Xiaoxiong Li, Yalei Li, Yong Wang, Yusen He, Guangyu Sun, Yamin Geng, Guoqing Chang

**Affiliations:** Technology Innovation Center of Modified Plastics of Hebei Province, College of Materials Science and Engineering, Hebei University of Engineering, Handan 056038, China

**Keywords:** sol–gel strategy, MXene/Fe_3_O_4_, cathode interlayer, lithium–sulfur batteries

## Abstract

Lithium–sulfur (Li-S) batteries are renowned for their high theoretical energy density and low cost, yet their practical implementation is hampered by the polysulfide shuttle effect and sluggish redox kinetics. Herein, a sol–gel strategy is proposed to engineer a multifunctional MXene/Fe_3_O_4_ composite as an efficient mediator for the cathode interlayer. The synthesized composite features Fe_3_O_4_ nanospheres uniformly anchored on the highly conductive Ti_3_C_2_T_x_ MXene lamellae, forming a unique 0D/2D conductive network. This structure not only provides abundant polar sites for strong chemical adsorption of polysulfides but also significantly enhances charge transfer, thereby accelerating the conversion kinetics. As a result, the Li-S battery based on the MXene/Fe_3_O_4_ interlayer delivers a high initial discharge capacity of 1367.1 mAh g^−1^ at 0.2 C and maintains a stable capacity of 1103.4 mAh g^−1^ after 100 cycles, demonstrating an exceptionally low capacity decay rate of only 0.19% per cycle. Even at a high rate of 1 C, a remarkable capacity of 1066.1 mAh g^−1^ is retained. Electrochemical analyses confirm the dual role of the composite in effectively suppressing the shuttle effect and catalyzing the polysulfide conversion. This sol–gel engineering approach offers valuable insight into the design of high-performance mediators for advanced Li-S batteries.

## 1. Introduction

Lithium–sulfur batteries (LSBs) are regarded as promising candidates for next-generation energy storage systems due to their high theoretical energy density (2600 Wh kg^−1^) and specific capacity (1675 mAh g^−1^), along with the natural abundance and environmental compatibility of sulfur [1,2,3,4,5,6,7]. However, their commercialization is impeded by several intrinsic challenges, including the insulating nature of sulfur and its discharge products (Li_2_S/Li_2_S_2_), the severe shuttling of soluble lithium polysulfides (LiPSs), and significant volume expansion during cycling. These issues result in rapid capacity fading, low Coulombic efficiency, and limited cycle life, necessitating the development of advanced functional materials to address these drawbacks [8,9,10,11,12].

MXenes, a family of two-dimensional transition metal carbides and nitrides, have recently attracted considerable attention in energy storage applications. For instance, Ti_3_C_2_T_x_ MXene exhibits metallic conductivity, rich surface chemistry, and a layered structure that facilitates ion diffusion and electrolyte penetration. More importantly, the surface terminations (–O, –OH, –F) of MXenes enable strong polar interactions with LiPSs, effectively suppressing their shuttling [13,14,15]. Nevertheless, MXene nanosheets are prone to restacking, which reduces active surface area and impedes ion accessibility [16]. To overcome the limitations of MXenes while leveraging their conductive and interfacial properties, researchers have explored hybrid structures with metal oxides. Among these, transition metal oxides such as Fe_3_O_4_ have been widely employed as functional mediators in LSBs due to their strong chemisorption capability toward LiPSs and catalytic activity in accelerating LiPS conversion. The polar surfaces of Fe_3_O_4_ can effectively anchor polysulfides, while its semiconductor-like properties can be mitigated by compounding with conductive substrates [17,18,19,20]. However, the aggregation of Fe_3_O_4_ nanoparticles and their limited conductivity often hinder the overall electrochemical performance when used alone.

In this work, a sol–gel method is employed to engineer a MXene/Fe_3_O_4_ composite characterized by uniform dispersion of Fe_3_O_4_ nanoparticles on MXene nanosheets. This synthetic approach promotes intimate interfacial contact and yields a hierarchical architecture conducive to Li^+^ diffusion and effective polysulfide confinement. The resulting composite acts as a multifunctional mediator, integrating efficient LiPS trapping with accelerated redox kinetics. Notably, the structural advantages of such heterostructures are further evidenced by morphological analyses. The van der Waals-facilitated self-assembly process enables ideal interfacial arrangement between Fe_3_O_4_ and MXene, where both constituents exhibit a complementary configuration. This ideal arrangement not only enhances electrolyte accessibility but also strengthens the structural integrity of the electrode during cycling. By combining the respective advantages of MXene and Fe_3_O_4_, the proposed composite offers a viable pathway toward high-performance and long-lasting lithium–sulfur batteries. The rational design of interface-engineered heterostructures paves the way for advanced energy storage systems with enhanced kinetics and stability.

## 2. Results and Discussion

**Figure 1:**
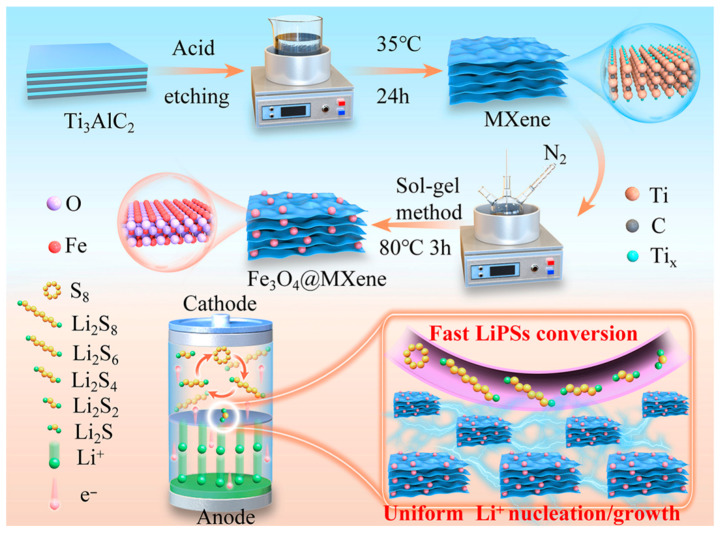
Schematic diagram of the synthesis mechanism of Fe_3_O_4_@MXene.

Figure 1 illustrates the schematic synthesis process of the Fe_3_O_4_@MXene composite and its proposed mechanism when used as a functional cathode in Li-S batteries. First, the layered Ti_3_AlC_2_ (MAX phase) precursor undergoes an acid etching process at 35 °C for 24 h to produce multilayer MXene sheets. The inset provides a magnified view of the MXene structure, depicting the layered arrangement of individual sheets and the atomic constituents (Ti, C, O). Subsequently, the obtained MXene is combined with Fe_3_O_4_ nanoparticles via a sol–gel method under a N_2_ atmosphere at 80 °C for 3 h, resulting in the final Fe_3_O_4_@MXene composite, where Fe_3_O_4_ nanoparticles are uniformly anchored on the MXene matrix. The Fe_3_O_4_@MXene composite is shown to facilitate fast lithium polysulfide (LiPS) conversion, as indicated by the sequential redox reactions from S_8_ and long-chain polysulfides (Li_2_S_8_, Li_2_S_6_, Li_2_S_4_) to short-chain species (Li_2_S_2_, Li_2_S). This process is mediated by the synergistic effect of the MXene conductive network and the Fe_3_O_4_ adsorption/catalytic sites. Simultaneously, the composite promotes uniform Li^+^ nucleation and growth on the anode side, ensuring a stable Li^+^ flux and electron (e^−^) transfer, which collectively contribute to suppressing the polysulfide shuttle effect and enhancing the battery’s redox kinetics.

**Figure 2:**
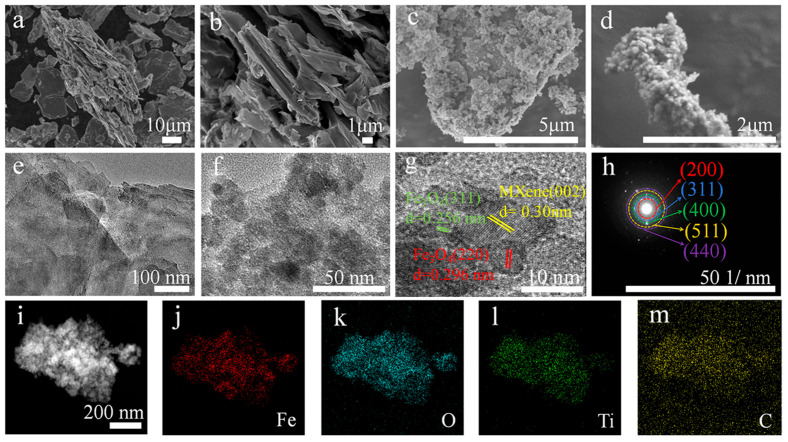
(**a**,**b**) SEM images of MXene. (**c**,**d**) SEM images of Fe_3_O_4_@MXene. (**e**) TEM image of MXene. (**f**) TEM image of Fe_3_O_4_@MXene. (**g**) HRTEM image of Fe_3_O_4_@MXene. (**h**) SAED pattern of Fe_3_O_4_@MXene. (**i**–**m**) Mapping images of Fe_3_O_4_@MXene.

Figure 2 presents a comprehensive microstructural and elemental analysis of the MXene and the Fe_3_O_4_@MXene composite. The scanning electron microscopy (SEM) image in Figure 2a reveals the intrinsic layered morphology of the MXene matrix. At higher magnification (Figure 2b), the accordion-like structure with interlayer spacing is clearly visible. After compositing with Fe_3_O_4_, the SEM images (Figure 2c,d) demonstrate that numerous Fe_3_O_4_ nanoparticles are uniformly anchored onto the MXene sheets, forming a robust hybrid architecture. Meanwhile, Figure S1a shows samples with partially uneven loading. The low-magnification transmission electron microscopy (TEM) image of MXene (Figure 2e) confirms its ultrathin two-dimensional sheet-like structure. The TEM image of the Fe_3_O_4_@MXene composite (Figure 2f) further verifies the close integration between the Fe_3_O_4_ nanoparticles and the MXene support. High-resolution TEM (HRTEM) analysis (Figure 2g) reveals distinct lattice fringes with measured spacings of 0.30 nm [21] and 0.256 nm, which are indexed to the (002) plane of MXene and the (311) plane of the spinel Fe_3_O_4_, respectively, confirming the successful formation of the heterostructure. The corresponding selected-area electron diffraction (SAED) pattern (Figure 2h) displays polycrystalline diffraction rings that can be ascribed to the crystal planes of Fe_3_O_4_, indicating its high crystallinity. Finally, high-angle annular dark-field scanning TEM (HAADF-STEM) and elemental mapping (Figure 2i–m) were performed to investigate the elemental distribution. The results confirm the homogeneous distribution of Iron (Fe, red), Oxygen (O, cyan), Titanium (Ti, green), and Carbon (C, yellow) throughout the composite, validating the uniform composition of the Fe_3_O_4_@MXene.

**Figure 3:**
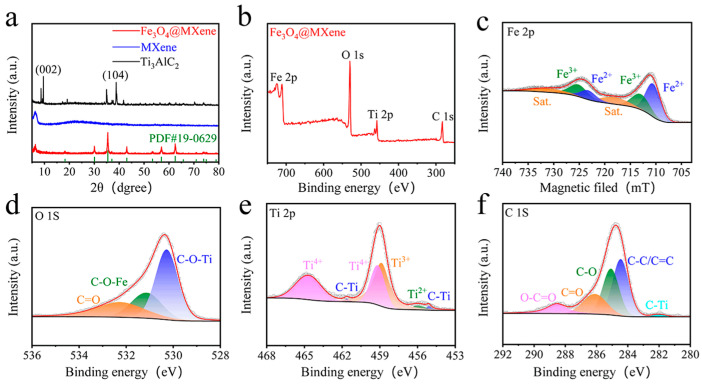
(**a**) XRD spectra of Fe_3_O_4_@MXene, MXene, and Ti_3_AlC_2_. (**b**) XPS survey spectrum of Fe_3_O_4_@MXene. (**c**) High-resolution XPS spectra of Fe 2p of Fe_3_O_4_@MXene. (**d**) High-resolution XPS spectra of O 1s of Fe_3_O_4_@MXene. (**e**) High-resolution XPS spectra of Ti 2p of Fe_3_O_4_@MXene. (**f**) High-resolution XPS spectra of C 1s of Fe_3_O_4_@MXene.

Figure 3a presents the X-ray diffraction (XRD) patterns of Fe_3_O_4_@MXene, MXene, and the precursor Ti_3_AlC_2_. All diffraction peaks for Fe_3_O_4_@MXene can be indexed to the inverse spinel structure of Fe_3_O_4_ (JCPDS No. 19-0629) [22]. The characteristic (002) peak of MXene is observed in both the MXene and Fe_3_O_4_@MXene samples, confirming the preservation of the MXene layered structure after compositing. The disappearance of the (104) peak associated with Ti_3_AlC_2_ indicates the successful etching of the Al layer to produce MXene. The XRD pattern of Fe_3_O_4_@MXene clearly shows the principal reflections of Fe_3_O_4_, demonstrating the successful formation of the composite. The XRD data indicate that the Fe_3_O_4_ grain size is approximately 30.69 nm, consistent with SEM observations (Figure S2). The average crystallite size of the Fe_3_O_4_ nanoparticles was estimated from XRD data using the Debye-Scherrer equation [23]:

$$D=\frac{K\lambda }{\beta \cos\theta }$$

where *D* is the average crystallite size (nm), *K* is the Scherrer constant (0.89), *λ* is the X-ray wavelength (0.15406 nm for Cu Kα), *β* is the full width at half maximum (FWHM, in radians) of the diffraction peak after instrument broadening correction, and *θ* is the Bragg diffraction angle.

The X-ray photoelectron spectroscopy (XPS) survey spectrum of Fe_3_O_4_@MXene (Figure 3b) confirms the presence of Fe, Ti, O, and C elements, consistent with the composite’s composition. High-resolution spectra were deconvoluted to investigate the chemical states. The Fe 2p spectrum (Figure 3c) exhibits two main spin–orbit doublets [24]. The peaks at binding energies of approximately 710.9 eV and 724.5 eV are assigned to Fe 2p_3_/_2_ and Fe 2p_1_/_2_ of Fe^3+^, respectively, while the peaks at 709.5 eV and 723.1 eV are characteristic of Fe^2+^, verifying the coexistence of both oxidation states in the inverse spinel Fe_3_O_4_ structure [25]. The O 1s spectrum (Figure 3d) can be fitted into three components. The peak at ~530.1 eV corresponds to metal-oxygen bonds (Fe-O, Ti-O), the peak at ~531.3 eV is attributed to oxygen in C=O and/or C-O-Ti/Fe bonds, indicating a strong interaction between Fe_3_O_4_ and the MXene substrate, and the component at higher binding energy (~532.5 eV) is associated with adsorbed water or surface hydroxyl groups [26]. The Ti 2p spectrum (Figure 3e) shows doublets for Ti^4+^ (e.g., Ti 2p_3_/_2_ at ~459.0 eV) and Ti^3+^ (Ti 2p_3_/_2_ at ~457.5 eV), confirming the presence of Ti-C bonds and the partially reduced nature of MXene [27]. The C 1s spectrum (Figure 3f) is deconvoluted into several peaks: C-C/C=C (284.8 eV), C-O (286.0 eV), and C-Ti (281.8 eV) [28,29]. The presence of the C-Ti bond is a distinctive fingerprint of MXene. The collective XPS analysis confirms the successful synthesis of the Fe_3_O_4_@MXene composite and reveals strong interfacial interactions, likely through Fe-O-Ti bonds. This bond facilitates rapid electron transfer from the conductive MXene substrate to the catalytic Fe_3_O_4_ sites, thereby dramatically accelerating the surface redox reactions of LiPSs. The interface itself may also create unique catalytic sites with optimized adsorption energy for polysulfides. This interface is crucial for enhancing the structural stability and electronic conductivity of the material [30,31].

**Figure 4:**
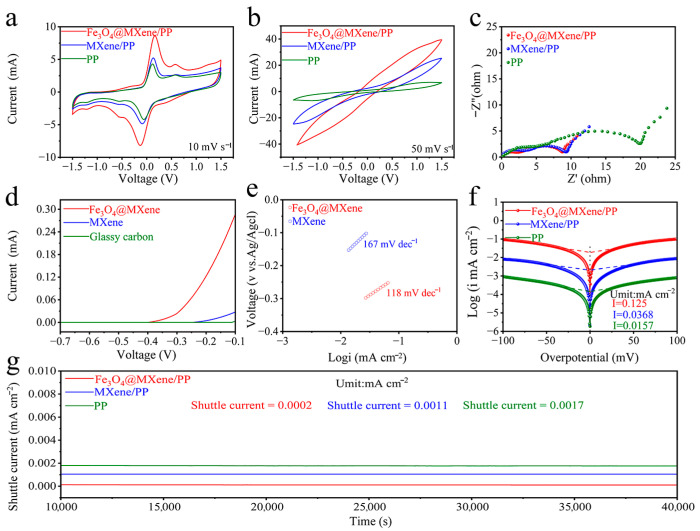
(**a**,**b**) CV curves of symmetric cells with Fe_3_O_4_@MXene/PP, Fe_3_O_4_/PP and PP. (**c**) EIS curves. (**d**) LSV curves of sulfide oxidation reaction on different catalysts. (**e**) Tafel plots calculated from LSV curves. (**f**) Tafel plots of Li_2_S_6_ symmetric cells. (**g**) Shuttle current plots of Fe_3_O_4_@MXene/PP, Fe_3_O_4_/PP and PP.

To systematically evaluate the electrocatalytic performance of Fe_3_O_4_@MXene/PP composites toward lithium polysulfides (LiPSs) in lithium–sulfur batteries, we assembled a symmetrical Li_2_S_6_ cell to investigate their redox kinetics. The relevant electrochemical data are shown in Figure 4. The cyclic voltammetry (CV) curves in Figure 4a,b, recorded at scan rates of 10 mV s^−1^ and 50 mV s^−1^, respectively, demonstrate that the cell with Fe_3_O_4_@MXene/PP electrodes exhibits the highest peak current intensity and the largest integrated area among the three samples (Fe_3_O_4_@MXene/PP, Fe_3_O_4_/PP, and PP). This indicates significantly enhanced redox reversibility and superior electrocatalytic activity for LiPS conversion. Electrochemical impedance spectroscopy (EIS) analysis (Figure 4c) reveals that the Fe_3_O_4_@MXene/PP-based cell exhibits the smallest charge-transfer resistance (R_ct_), suggesting facilitated electron transfer and faster reaction kinetics at the electrode/electrolyte interface. The electrocatalytic efficacy was further quantified by linear sweep voltammetry (LSV) for the sulfide oxidation reaction (Figure 4d). Compared to the unevenly loaded Fe_3_O_4_ sample’s electrocatalytic activity profile in Figure S1b, the uniformly loaded sample exhibits significantly superior performance. The Fe_3_O_4_@MXene catalyst shows a markedly lower onset potential compared to MXene and a glassy carbon electrode, confirming a reduced energy barrier for polysulfide conversion. The corresponding Tafel plot derived from the LSV data is presented in Figure 4e. The Fe_3_O_4_@MXene composite exhibits a Tafel slope of 118 mV dec^−1^, which is substantially lower than that of MXene (167 mV dec^−1^), underscoring its faster reaction kinetics. Furthermore, Tafel analysis of the Li_2_S_6_ symmetric cells (Figure 4f) yields consistent results, with Fe_3_O_4_@MXene showing the lowest Tafel slope, affirming its excellent catalytic activity. The exchange current density, a key kinetic parameter, was calculated to be the highest for Fe_3_O_4_@MXene, indicating the most favorable LiPS conversion kinetics. Finally, the shuttle effect was assessed by monitoring the steady-state current (Figure 4g). The cell employing the Fe_3_O_4_@MXene/PP separator displays a negligible shuttle current of only 0.0002 mA cm^−2^, which is drastically lower than that of Fe_3_O_4_/PP (0.0011 mA cm^−2^) and the pristine PP separator (0.0017 mA cm^−2^). This result provides direct evidence that the Fe_3_O_4_@MXene composite effectively suppresses the shuttling of soluble polysulfides. In summary, the collective electrochemical data unequivocally demonstrate that the Fe_3_O_4_@MXene/PP modifier serves as a highly efficient catalytic platform for accelerating LiPS redox kinetics and inhibiting the polysulfide shuttle, thereby contributing to the enhanced performance of Li-S batteries.

**Figure 5:**
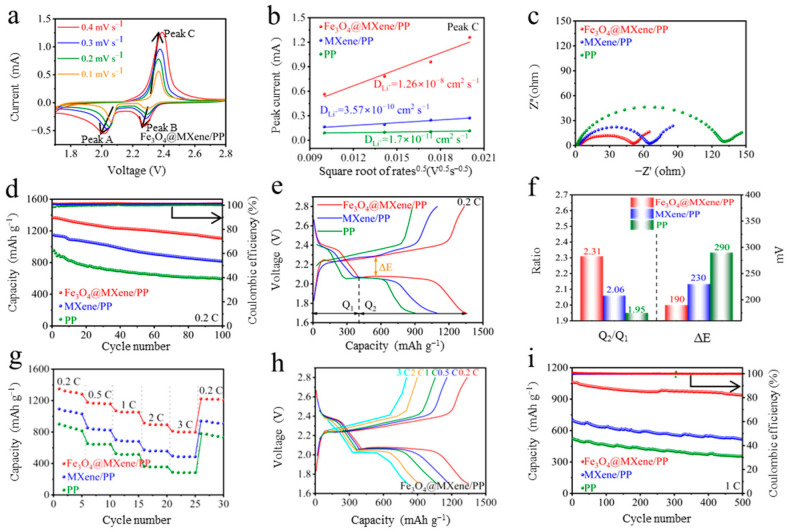
(**a**) CV curves of the Fe_3_O_4_@MXene/PP separator at different scan rates. (**b**) Peak current versus square root of scan rate. (**c**) EIS curves. (**d**) Cycling performance at 0.2 C. (**e**) GCD curves of different separators at 0.2 C. (**f**) Q_2_/Q_1_ values and ΔE derived from GCD curves. (**g**) Rate performance. (**h**) GCD curves of the Fe_3_O_4_@MXene/PP separator at different current densities and (**i**) cycling performance at 1C.

Figure 5 comprehensively evaluates the electrochemical performance of Li-S batteries employing the Fe_3_O_4_@MXene/PP composite as a functional separator. To evaluate the actual charge–discharge capacity of Fe_3_O_4_@MXene, each set of electrochemical experiments was conducted three times independently, and the average value was obtained [32]. Figure 5a presents the cyclic voltammetry (CV) curves of the cell with the Fe_3_O_4_@MXene/PP separator at scan rates ranging from 0.1 to 0.5 mV s^−1^. All curves exhibit two distinct cathodic peaks and one anodic peak, corresponding to the reduction of S_8_ to soluble long-chain LiPSs, followed by further reduction to insoluble Li_2_S_2_/Li_2_S, and the reverse oxidation process, respectively. The well-overlapping curves indicate excellent reaction reversibility. The linear relationship between the peak currents (Ip) and the square root of the scan rate (v^1^/^2^) is depicted in Figure 5b, suggesting that the redox reactions are controlled by Li^+^ diffusion. The calculated Li^+^ diffusion coefficient (D_Li_^+^) for the Fe_3_O_4_@MXene/PP cell is the highest among the compared systems, confirming accelerated reaction kinetics. Electrochemical impedance spectroscopy (EIS) analysis (Figure 5c) reveals that the cell with the Fe_3_O_4_@MXene/PP separator possesses the smallest resistance. To investigate the kinetics of electrode interface reactions in depth, we performed equivalent circuit fitting on the obtained electrochemical impedance spectra. All spectra were fitted using the equivalent circuit model shown in Figure S8, which consists of solution resistance (Rₛ), charge transfer resistance (R_ct_), ion diffusion resistance (W_o_), and constant phase element (CPE). The fitting results reveal that the Rct value of the Fe_3_O_4_@MXene electrode (50.43 Ω) is significantly lower than that of MXene (67.34 Ω), indicating faster charge transfer kinetics. This finding is highly consistent with the results from the cycling performance tests. The cycling stability at 0.2 C is shown in Figure 5d. The Fe_3_O_4_@MXene/PP cell delivers a high initial discharge capacity of 1367.1 mAh g^−1^ and maintains a capacity of 1103.4 mAh g^−1^ after 100 cycles, demonstrating superior capacity retention compared to cells with Fe_3_O_4_/PP and pristine PP separators. The galvanostatic charge–discharge (GCD) profiles at 0.2 C (Figure 5e) show that the Fe_3_O_4_@MXene/PP cell has the highest discharge capacity and the smallest voltage gap (ΔE) between the charge and discharge plateaus, signifying lower polarization and enhanced redox kinetics. A quantitative analysis of the GCD curves is summarized in Figure 5f. The Fe_3_O_4_@MXene/PP cell exhibits the highest Q_2_/Q_1_ ratio and the lowest ΔE value, underscoring its superior catalytic activity in promoting the solid–liquid conversion of LiPSs to Li_2_S_2_/Li_2_S, which is typically the rate-limiting step. The rate capability is illustrated in Figure 5g. The Fe_3_O_4_@MXene/PP cell achieves outstanding specific capacities of 1352.3, 1180.1, 1056.7, 955.4, and 890.1 mAh g^−1^ at current densities of 0.2, 0.5, 1, 2, and 3 C, respectively. When the current density is returned to 0.2 C, a high capacity of 1230 mAh g^−1^ is recovered, highlighting excellent reversibility. The corresponding GCD curves at different rates (Figure 5h) maintain well-defined voltage plateaus even at 3 C, indicating stable reaction kinetics under high-rate conditions. Finally, the long-term cycling performance at 1 C (Figure 5i) shows that the Fe_3_O_4_@MXene/PP cell maintains a high discharge capacity of 905.8 mAh g^−1^ after 300 cycles, with a capacity decay rate of only 0.058% per cycle and a nearly 99.8% Coulombic efficiency. These results collectively affirm that the Fe_3_O_4_@MXene/PP separator significantly enhances the sulfur utilization, reaction kinetics, and cycling stability of Li-S batteries. Characterized by transmission electron microscopy, the Fe_3_O_4_@MXene composite was found to exhibit outstanding structural stability after 500 cycles. As shown in Figure S11, the composite retains its original structure, with Fe_3_O_4_ nanoparticles uniformly and firmly anchored to MXene sheets. No nanoparticle desorption, aggregation, or significant oxidation of the MXene substrate was observed, providing direct evidence for the stability of the Fe_3_O_4_@MXene structure.

**Figure 6:**
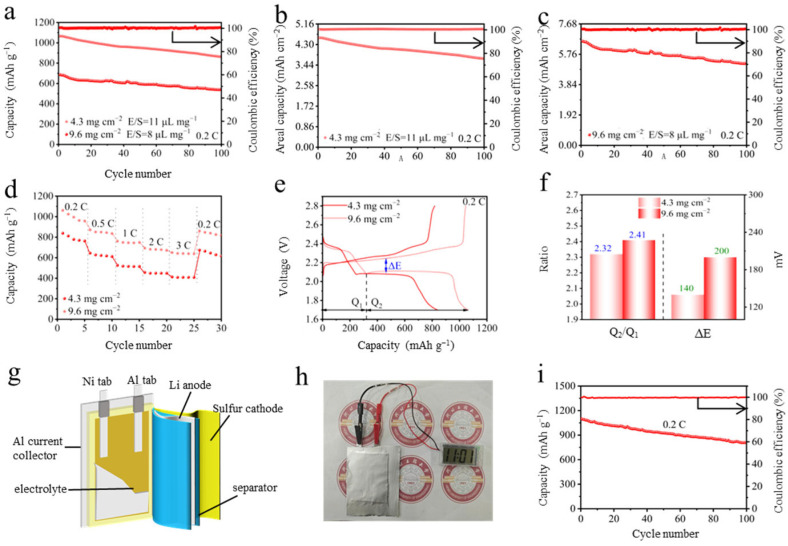
(**a**–**c**) Cycling performance with different sulfur loadings at 0.2 C. (**d**) Rate performance. (**e**) GCD curves with different sulfur loadings at 0.2 C. (**f**) Q2/Q1 values and ΔE derived from GCD curves. (**g**) schematic diagram of the Li-S pouch cell structure. (**h**) electronic watch lit by the Li-S pouch cell. (**i**) Cycling performance of Li-S pouch cell based on Fe_3_O_4_@MXene/PP separator at 0.2 C.

Figure 6 evaluates the practical application potential of the Fe_3_O_4_@MXene-based functional separator in Li-S batteries, particularly under high-sulfur-loading conditions. The long-term cycling stability at 0.2 C with different sulfur loadings is presented in Figure 6a–c. Impressively, even with a high sulfur loading of 9.6 mg cm^−2^, the battery delivers a high initial discharge capacity of 696.8 mAh g^−1^ and maintains excellent capacity retention after 100 cycles, demonstrating robust electrochemical stability. The rate performance under high sulfur loading is shown in Figure 6d. The cell exhibits remarkable capability, sustaining substantial specific capacities at current densities from 0.2 C to 3 C, which highlights the efficient reaction kinetics and superior sulfur utilization facilitated by the Fe_3_O_4_@MXene/PP separator. The GCD curves at 0.2 C with varying sulfur loadings are displayed in Figure 6e. All curves show two distinct discharge plateaus and one charge plateau, indicative of typical multi-step sulfur redox reactions. Notably, even at elevated sulfur loadings, the voltage polarization remains low, underscoring the catalytic effect of Fe_3_O_4_@MXene in promoting polysulfide conversion. A quantitative analysis derived from the GCD curves (Figure 6f) reveals a high Q_2_/Q_1_ ratio and a minimal voltage gap (ΔE), confirming the material’s efficacy in enhancing the kinetics of the solid-phase precipitation reaction (Li_2_S_2_/Li_2_S). To demonstrate practical applicability, a schematic illustration of the assembled Li-S pouch cell structure is shown in Figure 6g. The successful implementation of this technology is visually confirmed in Figure 6h, where a commercial electronic watch is reliably powered by the Fe_3_O_4_@MXene/PP-based pouch cell. Finally, the cycling performance of the practical pouch cell at 0.2 C is quantified in Figure 6i. The cell achieves a high initial discharge capacity of 1132.5 mAh g^−1^ and maintains favorable capacity retention over 100 cycles, providing compelling evidence for the feasibility of using the Fe_3_O_4_@MXene composite in high-performance, practical Li-S batteries. Collectively, the data in Figure 6 validate the significant promise of the Fe_3_O_4_@MXene/PP separator for enabling Li-S batteries with high energy density and practical viability.

## 3. Conclusions

In summary, a facile sol–gel strategy was successfully employed to engineer a multifunctional MXene/Fe_3_O_4_ composite as an efficient mediator for Li-S batteries. This approach enables the uniform anchoring of Fe_3_O_4_ nanospheres onto the highly conductive MXene lamellae, forming a unique 0D/2D hierarchical architecture. Within this configuration, the MXene substrate serves as an excellent electron conductor, facilitating rapid charge transfer, while the evenly dispersed Fe_3_O_4_ nanospheres provide abundant polar sites for strong chemical adsorption of lithium polysulfides. This synergistic interaction not only effectively suppresses the polysulfide shuttle effect but also significantly accelerates the sulfur redox kinetics. As a result, the Li-S battery incorporating the MXene/Fe_3_O_4_ composite achieves an impressive initial discharge capacity of 1367.1 mAh g^−1^ at 0.2 C and maintains a stable capacity of 1103.4 mAh g^−1^ after 100 cycles, corresponding to an exceptionally low capacity decay rate of only 0.19% per cycle. Even under high-rate conditions at 1 C, a remarkable capacity of 1066.1 mAh g^−1^ is retained. These findings demonstrate the dual functionality of the composite in polysulfide confinement and conversion catalysis, establishing this sol–gel engineered MXene/Fe_3_O_4_ composite as a highly promising mediator for advanced high-energy-density Li-S batteries.

## 4. Materials and Methods

### 4.1. Materials

Ferrous chloride tetrahydrate (FeCl_2_·4H_2_O, analytical grade) was purchased from Tianjin Ouboke Chemical Co., Ltd. (Tianjin, China) Ammonia (NH_4_OH, 25%) was purchased from Shanghai Maclean Co., Ltd. (Shanghai, China) Lithium fluoride (LiF, analytical grade) and ferric chloride hexahydrate (FeCl_3_·6H_2_O, analytical grade) were purchased from Aladdin Reagent Co., Ltd. (Shanghai, China) Mxene phase (Ti_3_AlC_2_, 400 mesh) was purchased from Jilin 11 Technology Co., Ltd. (Changchun, China).

### 4.2. Preparation

Preparation of Few-Layer MXene Dispersion: Lithium fluoride (1.2 g) and hydrochloric acid (9 M, 40 mL) were mixed in a polytetrafluoroethylene (PTFE) beaker and stirred for 30 min. Subsequently, MAX-phase Ti_3_AlC_2_ powder (2 g) was slowly added to the solution, and the mixture was stirred at 35 °C for 24 h to complete the etching process. The resulting suspension was centrifuged at 3500 rpm for 10 min, and the supernatant was discarded. The residue was repeatedly washed with deionized water and sonicated for 10 min until the pH of the supernatant reached approximately 5. The obtained sediment was then dispersed in 40 mL of ethanol and sonicated for 1 h to facilitate intercalation, followed by centrifugation at 10,000 rpm for 10 min to collect the precipitate. Subsequently, the precipitate was redispersed in 20 mL of deionized water, sonicated for 20 min, and centrifuged at 3500 rpm for 3 min. The dark brown supernatant was collected as the few-layer MXene dispersion. This step was repeated several times to obtain additional few-layer dispersions.

Preparation of Fe_3_O_4_@MXene by sol-gel method: 0.2 g of MXene powder was ultrasonically dispersed in 40 mL of deionized water under nitrogen protection for 30 min. Subsequently, 0.530 g of FeCl_2_·4H_2_O and 1.08 g of FeCl_3_·6H_2_O were added to the MXene dispersion and magnetically stirred for 30 min under a continuous nitrogen atmosphere. The mixture was then transferred to a three-necked flask placed in an oil bath and heated to 80 °C under nitrogen with constant stirring. When the temperature approached 80 °C, 25% aqueous ammonia solution was added dropwise in small portions until the pH of the mixture reached 10. The reaction was continued for 3 h to ensure complete formation of Fe_3_O_4_ nanoparticles. After cooling to room temperature, the product was collected with a magnet, thoroughly washed with deionized water, and then freeze-dried to obtain Fe_3_O_4_@MXene composite powder.

### 4.3. Assembling the Battery

In an argon-filled glove box (oxygen and water content < 0.1 ppm), CR2032 coin cells were assembled using a S/CNTs composite cathode and a lithium metal anode as electrodes, with polypropylene (PP) separators, Mxene/PP separators, and Fe_3_O_4_@Mxene/PP separators, respectively. The electrolyte consisted of 1.0 M LiTFSI and 2 wt.% LiNO_3_ dissolved in a DME:DOL (volume ratio 1:1) mixture. Electrochemical characterization was performed after 12 h of quiescence.

### 4.4. Material Characterization

The morphology of the prepared materials was characterized by field emission scanning electron microscopy (FESEM) using a NOVA NanoSEM 450 microscope (FEI Company, Hillsboro, OR, USA) and transmission electron microscopy (TEM) on an FEI Tecnai F20 instrument (FEI Company, Hillsboro, OR, USA). X-ray diffraction (XRD) measurements were performed on a SmartLab SE X-ray diffractometer (Rigaku Corporation, Tokyo, Japan). The surface elemental composition was analyzed by X-ray photoelectron spectroscopy (XPS) on a Thermo Scientific K-Alpha spectrometer (Thermo Fisher Scientific, Waltham, MA, USA).

### 4.5. Electrochemical Properties

The electrochemical performance of the Li–S batteries was tested using CR2032 coin cells. A working electrode was prepared by uniformly mixing S/CNTs, Ketjen black (conductive agent), and polytetrafluoroethylene (PVDF, binder) in N-methyl-2-pyrrolidone (NMP) at 70%, 20%, and 10% by mass, respectively. The mixed slurry was evenly coated on aluminum foil and then dried at 60 °C for 12 h. CR2032 coin cells were assembled using the prepared working electrode as the positive electrode, metallic lithium as the negative electrode, PP separators, MXene/PP separators, and Fe_3_O_4_@MXene/PP separators as separators, and an electrolyte consisting of 1.0 M LiTFSI and 2 wt.% LiNO_3_ dissolved in a 1:1 (volume ratio) mixture of DME and DOL. The cyclic voltammetry (CV) test used a CHI660E electrochemical workstation (Cosit Instrument Co., Ltd., Hangzhou, China) with a voltage window of 1.7–2.8 V. The constant current discharge/charge (GCD) test used a Newwei high-performance battery detection system with a voltage window of 1.7–2.8 V. The electrochemical impedance spectroscopy (EIS) test used a CHI660E electrochemical workstation with a frequency range of 10^−2^ to 10^5^ Hz.

## Data Availability

The original contributions presented in this study are included in the article/Supplementary Materials. Further inquiries can be directed to the corresponding author.

## Supplementary Materials

The following supporting information can be downloaded at: https://www.mdpi.com/article/10.3390/gels11120959/s1. Figure S1: (a) SEM images of synthesized Fe3O4@MXene and (b) Its LSV curves; Figure S2: (a) SEM images of synthesized Fe_3_O_4_@MXene and (b) their particle size distributions; Figure S3: Rate performance of Li||Li symmetric cells with Fe_3_O_4_@MXene/PP, Fe_3_O_4_/PP and PP separators; Figure S4: Cycling performance of Li||Li symmetric cells with Fe_3_O_4_@MXene/PP, Fe_3_O_4_/PP and PP separators; Figure S5: (a) Schematic diagram of lithium dendrites after cycling using Fe_3_O_4_@MXene/PP and PP separators. (b) SEM images of Fe_3_O_4_@MXene/PP, Fe_3_O_4_/PP and PP separators cycled lithium anodes; Figure S6: CV curves of the MXene/PP separator at different scan rates; Figure S7: CV curves of the PP separator at different scan rates; Figure S8: Nyquist diagrams and the fitted equivalent circuit of the cells; Figure S9: (a) XPS image of Fe_3_O_4_@MXene synthesized at 90 °C (b) XPS image of Fe_3_O_4_@MXene synthesized at 70 °C (c) Cycle performance at 90°C (d) 70 °C; Figure S10: Cycling Performance of Batteries with gradient Fe_3_O_4_@MXene coating (thicker near cathode, thinner near anode); Figure S11: TEM image of Fe_3_O_4_@MXene after 500 cycles; Figure S12: Electronic watch lit by the Li-S pouch cell under (a) lateral bending by 180° and (b) longitudinal bending by 180°.
