# Supplementary material for: Sol–Gel Engineered MXene/Fe3O4 as an Efficient Mediator to Suppress Polysulfide Shuttling and Accelerate Redox Kinetics
Source: Gels. 2025 Nov 28;11(12):959. doi: 10.3390/gels11120959 (PMC12732716; doi:10.3390/gels11120959)
Supplement: Supplementary file 1 [file gels-11-00959-s001.zip › gels-3989062-supplementary.pdf]

Supplementary Materials

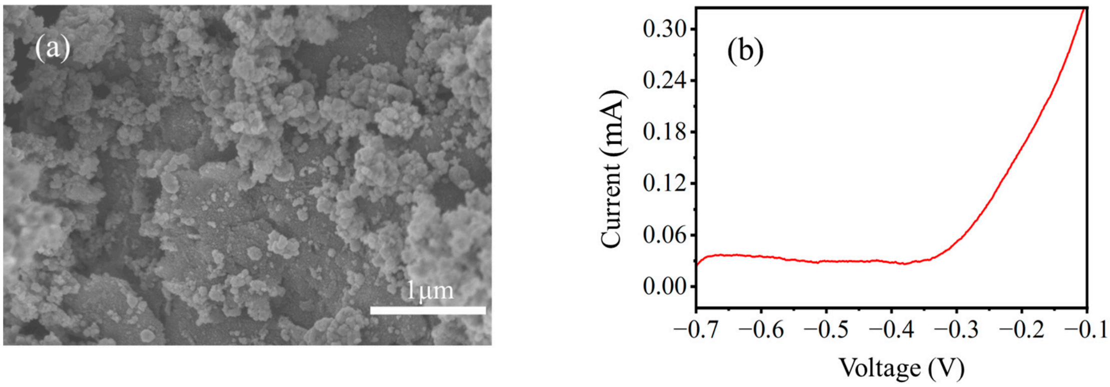

Figure S1. (a) SEM images of synthesized Fe_3O_4@MXene and (b) Its LSV curves

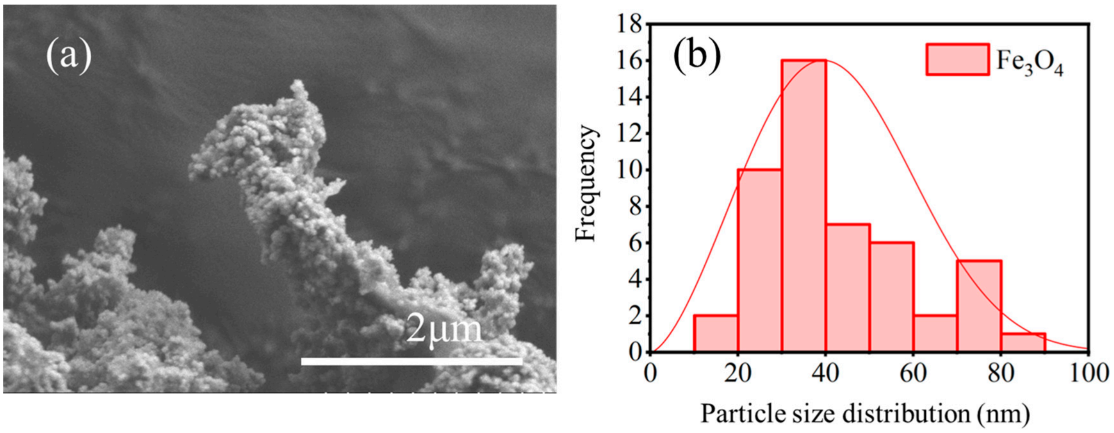

Figure S2. (a) SEM images of synthesized Fe_3O_4@MXene and (b) their particle size distributions

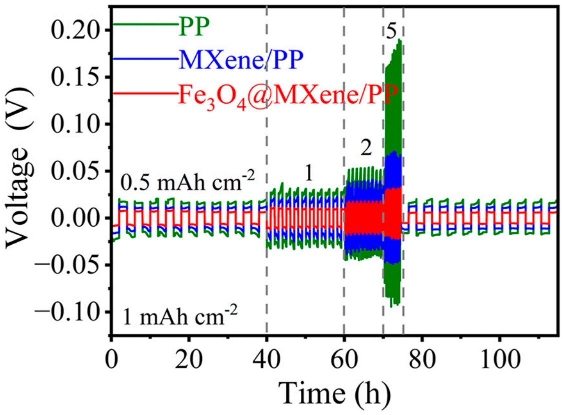

Figure S3. Rate performance of Li||Li symmetric cells with Fe_3O_4@MXene/PP, Fe_3O_4/PP and PP separators

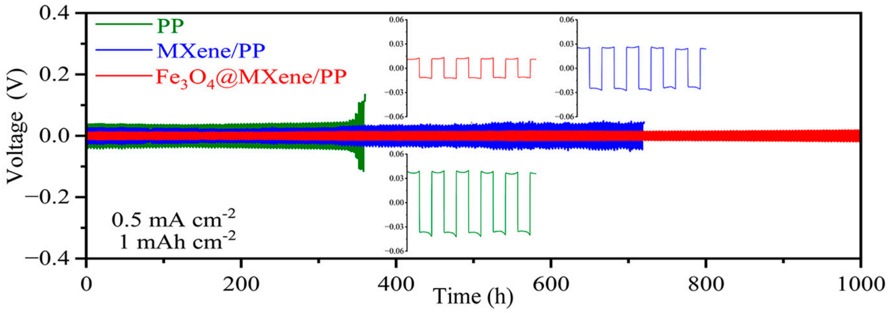

Figure S4. Cycling performance of Li||Li symmetric cells with Fe_3O_4@MXene/PP, Fe_3O_4/PP and PP separators.

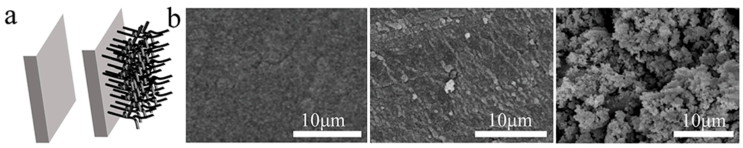

Figure S5. (a) Schematic diagram of lithium dendrites after cycling using Fe_3O_4@MXene/PP and PP separators. (b) SEM images of Fe_3O_4@MXene/PP, Fe_3O_4/PP and PP separators cycled lithium anodes.

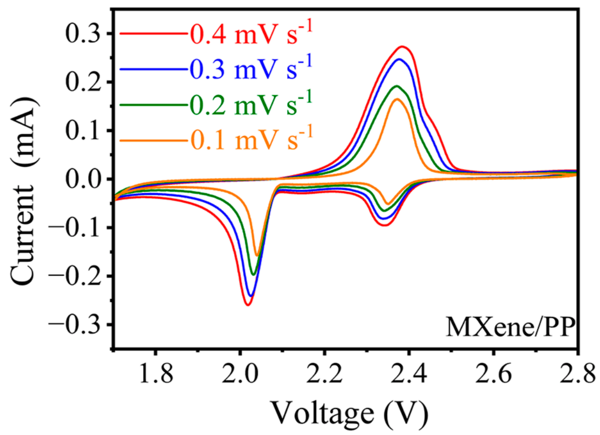

Figure S6. CV curves of the MXene/PP separator at different scan rates.

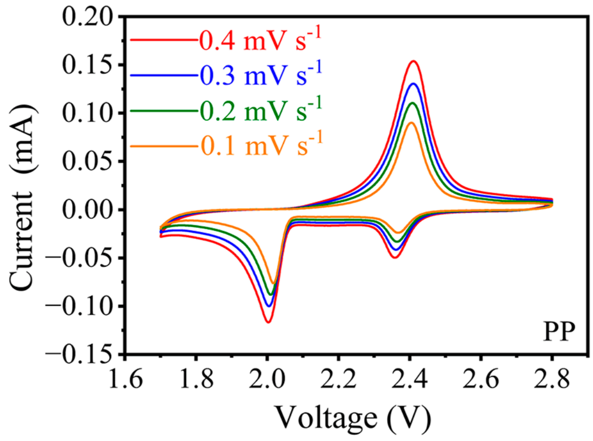

Figure S7. CV curves of the PP separator at different scan rates.

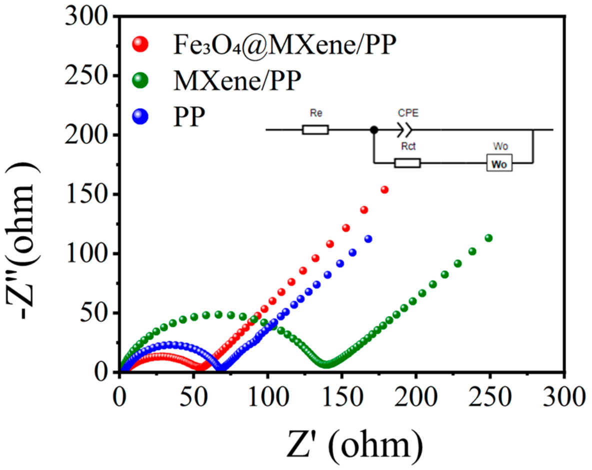

Figure S8. Nyquist diagrams and the fitted equivalent circuit of the cells.

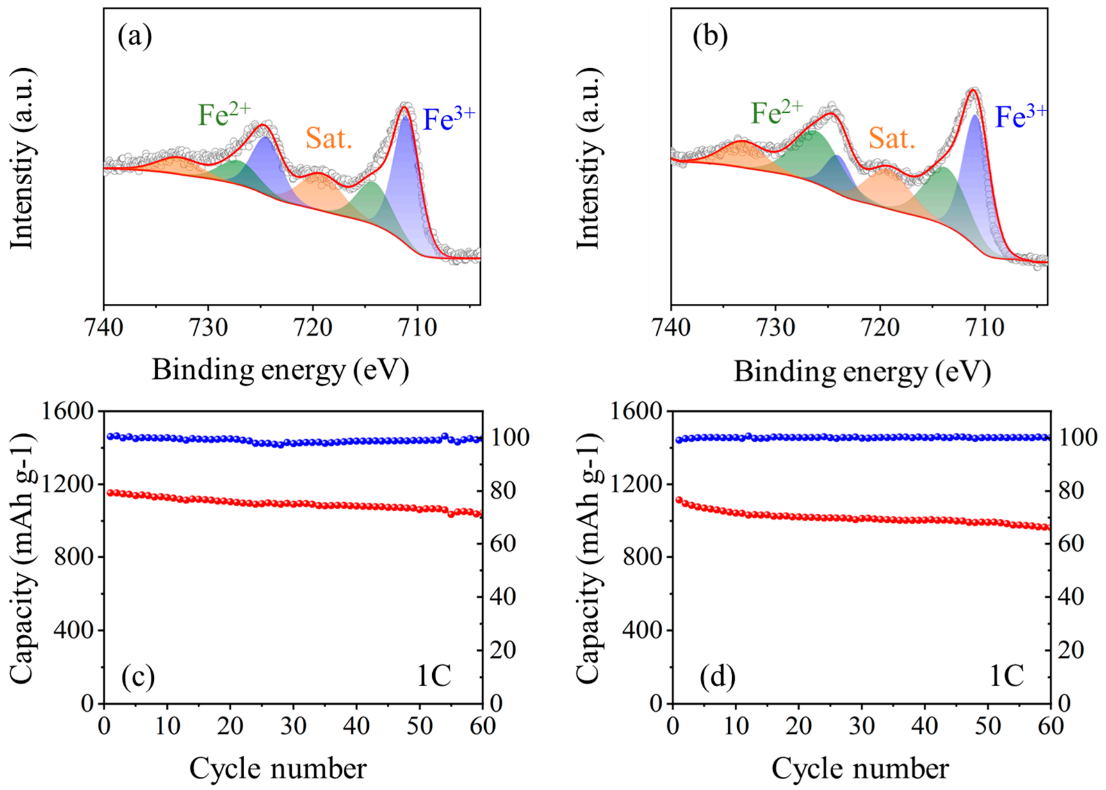

Figure S9. (a) XPS image of Fe_3O_4@MXene synthesized at 90 °C (b) XPS image of Fe_3O_4@MXene synthesized at 70 °C (c) Cycle performance at 90°C (d) 70 °C

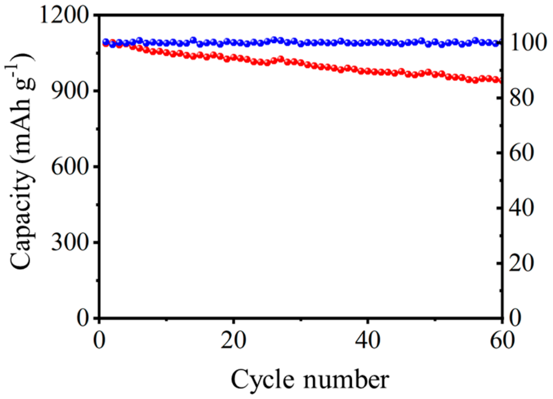

Figure S10. Cycling Performance of Batteries with gradient Fe_3O_4@MXene coating (thicker near cathode, thinner near anode)

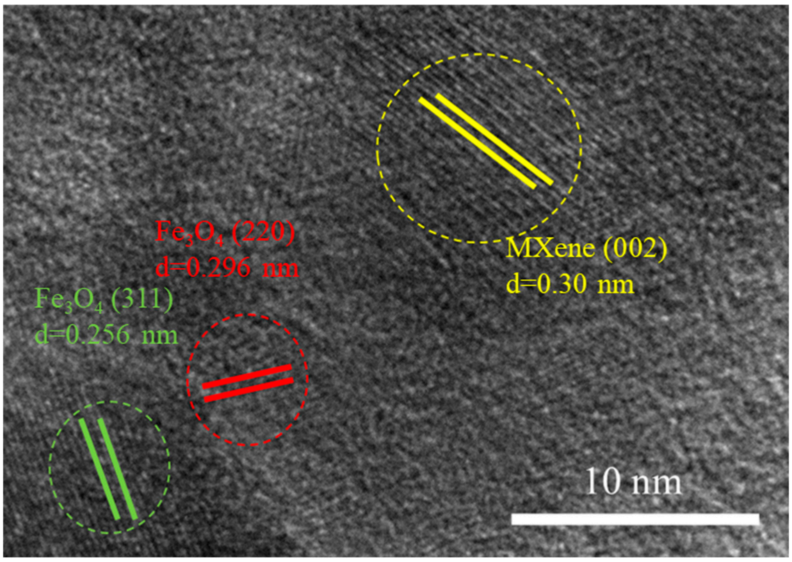

Figure S11. TEM image of Fe_3O_4@MXene after 500 cycles

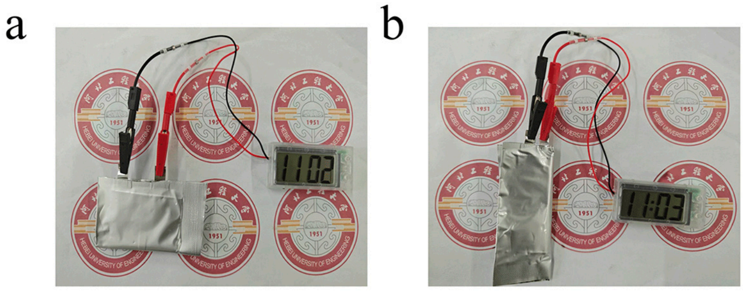

Figure S12. Electronic watch lit by the Li-S pouch cell under (a) lateral bending by 180° and (b) longitudinal bending by 180°.
